# Sphingosine-1-phosphate is a new biomarker for severity in human sepsis

**DOI:** 10.1186/cc14134

**Published:** 2015-03-16

**Authors:** MS Winkler, A Nierhaus, E Mudersbach, M Holzmann, A Bauer, L Robbe, C Zahrte, G Daum, S Kluge, C Zoellner

**Affiliations:** 1 University Medical Center Eppendorf, Hamburg, Germany; 2 University Heart Center, Hamburg, Germany

## Introduction

During sepsis a leading symptom is capillary leakage caused by endothelial damage, followed by multiorgan dysfunction. Sphingosine-1-phosphate (S1P) is a bioactive lipid with multiple functions. Cellular reactions depend on the S1P concentration in the blood and its binding to five specific G-protein coupled receptors. S1P-regulated functions include: control of endothelial permeability; lymphocyte migration across microvessels depending on an S1P gradient; and control of vascular tone [1]. This clinical study will address the question of whether S1P blood concentrations are associated with sepsis severity.

## Methods

Following ethical approval we enrolled patients fulfilling the ACCP/SCCM sepsis criteria into three groups (Group A: sepsis; Group B: severe sepsis; Group C: septic shock). A group of 20 healthy donors served as controls. Serum blood samples, laboratory data and clinical parameters are presented for day 1. The primary outcome variable was serum S1P concentration (μg/l) quantified by mass spectrometry (Agilent^®^). The SOFA score was used to describe disease severity.

## Results

We included 87 patients (32 Group A, 25 Group B, 30 Group C). The serum concentration of S1P (mean ± SD) in the control group was 484.6 ± 152.6 μg/l and significantly higher compared with Group A 239.4 ± 61.3 μg/l, Group B 248.6 ± 93.7 μg/l and Group C 141.6 ± 46.3 μg/l. We observed a negative correlation between S1P and SOFA score (Pearson *r *= -0.45, *P *< 0.001, *R*^2 ^= 0.2). The median SOFA score in our cohort was 6. We divided the cohort into two groups: SOFA score <6; and SOFA score >6. We tested the sensitivity and specificity of S1P to indicate disease severity by ROC analysis. In our cohort the area under the curve (AUC) for S1P was 0.77 (CI 0.670 to 0.870) and therefore higher when compared with common markers of inflammation (PCT, IL-6, CRP with AUC of 0.68 (0.560 to 0.796), 0.68 (0.554 to 0.786) and 0.67 (0.571 to 0.794), resp.).

## Conclusion

Our findings suggest that S1P is a novel marker for severity of sepsis with severe sepsis and septic shock being associated with low levels of S1P. Moreover, blood concentrations of S1P might play a key role in sepsis pathophysiology.

## Acknowledgements

MSW and AN are equal contributors.

## References

[B1] Maceyka M Sphingosine-1-phosphate signaling and its role in disease Trends Cell Biol 2012 22 50 60 10.1016/j.tcb.2011.09.003 22001186 PMC3253987

